# SOPROLIFE System: An Accurate Diagnostic Enhancer

**DOI:** 10.1155/2014/924741

**Published:** 2014-10-21

**Authors:** Mona Zeitouny, Mireille Feghali, Assaad Nasr, Philippe Abou-Samra, Nadine Saleh, Denis Bourgeois, Pierre Farge

**Affiliations:** ^1^Department of Aesthetic and Restorative Dentistry, Dental School, Lebanese University, Hadath, Lebanon; ^2^Faculty of Nursing and Health Sciences, Notre Dame University, Beirut, Lebanon; ^3^Department of Public Health, Faculty of Odontology, University of Lyon 1, 69008 Lyon, France; ^4^Department of Endodontics and Dentistry, Faculty of Odontology, University of Lyon 1, 69008 Lyon, France

## Abstract

*Objectives.* The aim of this study was to evaluate a light-emitting diode fluorescence tool, the SOPROLIFE light-induced fluorescence evaluator, and compare it to the international caries detection and assessment system-II (ICDAS-II) in the detection of occlusal caries. Methods. A total of 219 permanent posterior teeth in 21 subjects, with age ranging from 15 to 65 years, were examined. An intraclass correlation coefficient (ICC) was computed to assess the reliability between the two diagnostic methods. *Results.* The results showed a high reliability between the two methods (ICC = 0.92; IC = 0.901–0.940; *P* < 0.001). The SOPROLIFE blue fluorescence mode had a high sensitivity (87%) and a high specificity (99%) when compared to ICDAS-II. *Conclusion.* Compared to the most used visual method in the diagnosis of occlusal caries lesions, the finding from this study suggests that SOPROLIFE can be used as a reproducible and reliable assessment tool. At a cut-off point, categorizing noncarious lesions and visual change in enamel, SOPROLIFE shows a high sensitivity and specificity. We can conclude that financially ICDAS is better than SOPROLIFE. However SOPROLIFE is easier for clinicians since it is a simple evaluation of images. Finally in terms of efficiency SOPROLIFE is not superior to ICDAS but tends to be equivalent with the same advantages.

## 1. Introduction

Dental caries is a preventable and reversible infectious disease process [1, 2] to which people are susceptible throughout their lifetime [2]. Despite the benefits of its prevention through fluorides, toothpastes, sealants, improvements in diet, oral health education, and dental care [3], dental caries still remains a major problem worldwide [4] affecting 60–90% of schoolchildren and the vast majority of adults [5, 6]. Its prevalence is around 80% worldwide [7]. Molars and premolars are the most vulnerable teeth of caries attack related to the morphology of their occlusal surfaces [8] and the difficulty of plaque removal [9]. Many dentists continue to intervene when caries are still at enamel level [10]. For that reason, accurate preoperative diagnosis of caries depths and early occlusal caries detection are important to establish adequate preventive measures and avoid premature tooth treatment by restoration [9].

To date, there are two major techniques aimed at helping clinicians in detecting caries on occlusal surfaces [11] represented by visual examination and by light-based caries diagnostic tools as fiber optic transillumination (FOTI), DIAGNODent tool (KaVo), and SOPROLIFE. Visual examination of caries has progressed by establishing the international caries detection and assessment system (ICDAS) [12]; indeed, ICDAS-II, the second version, was improved and developed to provide a standardized system [13] to enable clinicians to diagnose and detect the first visual change in enamel leading to better information for clinical management [14, 15].

All diagnostic tools for detection and quantification of dental caries have to obey safety regulations, detect and differentiate shallow and deep lesions, and make monitoring possible by taking precise and quantitative measurement; in addition they have to be cost-effective and user-friendly [13].

The principle of FOTI is used since the seventies [16]. This technique uses a narrow beam of bright white light that is directed across areas of contact between the proximal surfaces and the disruption of crystal structure that deflects the light beam and thus produces shadows [1]. The DIAGNODent tool is based on laser fluorescence and detects porphyrins involvement areas; it appears to measure caries lesion rather than crystalline demineralization [17]. SOPROLIFE is a more recently released device using a light-induced fluorescence evaluator for diagnostic and treatment (LIFE D.T); it was developed and based on the imaging and autofluorescence of dental tissues [18, 19].

Till now, no study has looked at the reproducibility of the SOPROLIFE in the detection and assessment of occlusal caries. Therefore we designed a clinical study with the aim of evaluating the clinical sensitivity and specificity rates of SOPROLIFE as opposed to ICDAS for the detection of initial occlusal caries in noncavitated enamel in permanent premolars and molars.

## 2. Materials and Methods

### 2.1. Sample Patients' Selection

This study was conducted over 2 months from March 7 to May 10, 2013. Twenty-one patients were randomly selected (based on their arrival order) from all patients attending the Aesthetic and Restorative Dentistry Department of the Dental School of Lebanese University. Inclusion criteria were age between 15 and 65 years, with no gender restriction, and patients with fully unrestored dental arches.

Exclusion criteria were patients with posterior restorations on molars or premolars or poor oral health with chronic or acute dental infection. In addition patients with a significant past or current medical problem history were not considered for the study, that is, patients with conditions that may affect oral health or oral flora (i.e., diabetes, HIV, and heart conditions which require antibiotic prophylaxis) or taking medication that may affect the oral flora or salivary flow; pregnant or breastfeeding women were also excluded. The subjects who met the criteria were informed of the purpose of the study and verbal consent from the patient was obtained before the examination session.

Examiners start evaluating patients using the ICDAS. After finishing all the samples, they did the work using SOPRO. One should note that patients ID was hidden when working with SOPRO.

### 2.2. Observers

Two independent dentists (Mona Zeitouny and Mireille Feghaly) specialized in restorative and aesthetic dentistry randomly examined each tooth by two different methods of caries assessments consecutively and without knowing the results of each method: the visual examination and assessment using the ICDAS-II criteria (Table 1) [20] and the use of the light fluorescence device SOPROLIFE (SOPRO, ACTEON Group, La Ciotat, France). This method involves an intraoral LED light-emitting diode camera offering the ability to detect and locate differences in density, structures, and/or chemical composition of a biological tissue.

Twenty days prior to the initiation of the study, calibration sessions were arranged for the 2 operators and the two methods separately in the examination site. Observers were trained using 100 premolars and molars cleaned without sealants or restorations. Each observer examined each tooth and noted the results. Then, the observers compared the results between them and reviewed the discrepancy cases for calibration until the two observers reach a full concordance rate.

### 2.3. Tooth Cleaning

Before examination, the occlusal surfaces of each tooth were cleaned for 10 seconds with a water powder jet cleaner and sodium bicarbonate powder (EMS) (ProphyFlex II, KaVo and Biberach; Germany) and then rinsed by an air water spray for another 10-second period in order to remove any powder remnants from the fissure. Following this preparation step of the tooth surfaces, all examinations were conducted under standard conditions in a professional dental light with a front-surface dental mirror and an oil-free air syringe for drying teeth during 5 seconds. The drying procedure is a requisite both for the ICDAS-II evaluation and for the use of the SOPRO device.

### 2.4. Visual Examination

The visual examination was performed using the ICDAS-II criteria, which provides a standardized method of lesion detection. The ICDAS-II detection codes for coronal caries range from 0 to 6 depending on the severity of the lesion with the corresponding clinical views (Table 1).

In this study, we used the SOPROLIFE light-induced fluorescence evaluator system (SOPRO, ACTEON Group, La Ciotat, France) operating in the blue fluorescence mode, in which the system uses four white LEDs, and the magnification mode I with the disposable intraoral protection sheets and the intraoral tip. The blue LED, selected by the device, emits at a 450 nm-wavelength which excites a light fluorescence signal re-transmitted by dentine. The spectrum of the fluorescence signal is green when the dentine is healthy and dark red when the dentine is infected (according to the SOPROLIFE manufacturer's instructions). The images were recorded with the SOPRO IMAGING software. An HP 620 Notebook was used to collect the data.

When evaluating occlusal fissure areas in the SOPROLIFE blue fluorescence mode, we used the SOPROLIFE blue fluorescence mode score description as presented in Table 2.

Code 0 was given when the fissure appears shiny green, the enamel appears sound, and there are no visible changes. Code 1 was selected if a tiny, thin red shimmer in the pits and fissure system is observed, which can slightly come up the slopes (walls) of the fissure system. No red dots appeared. At code 2, darker red spots confined to the fissure are visible. For code 3 dark red spots have extended as lines into the fissure areas but remain confined to the fissures. A slight beginning roughness of the more lined red areas can be visible. If the dark red (or red-orange) extends wider than the confines of the fissures, code 4 was given. Code 5 was selected if obvious openings of enamel were seen with visible dentin [13].

### 2.5. Data Collection

Each tooth was evaluated and scored for lesions severity using a seven-category scale (0–6) according to ICDAS-II and a six-category scale (0–5) according to SOPROLIFE.

### 2.6. Statistical Analysis

Data were analyzed using the SPSS program (version 17, SPSS Inc., Chicago, IL, USA). In all analyses, a *P* value < 0.05 was considered significant. Demographic data are presented as mean ± one standard deviation (SD). Interobserver reproducibility with each examination method and between methods was assessed using intraclass correlation coefficients (ICCs). ICC values equal to 0 represent agreement equivalent to that expected by chance, while 1 represents full agreement. ICC values between 0 and 0.2 indicate poor agreement, values between 0.3 and 0.4 indicate fair agreement, values between 0.41 and 0.6 indicate moderate agreement, values between 0.61 and 0.8 indicate strong agreement, and values greater than 0.8 indicate almost perfect agreement. In addition, a Bland and Altman analysis was done to show graphically the difference between the two methods. Sensitivity and specificity of the new diagnostic system for detecting caries in noncavitated lesions were calculated by reference to the ICDAS-II values. Since we are interested in noncavitated lesions, the calculation was made by dividing scores of the two diagnostic methods into two groups: group 1 which included scores 0 representing healthy teeth without caries and group 2 which included scores 1 and 2 both representing visual change in enamel.

## 3. Results

This study compared the SOPROLIFE device (in blue fluorescence mode) to the ICDAS-II in the detection of caries lesions. Twenty-one patients were evaluated in this study and a total of 219 teeth (98 permanent molars and 121 permanent premolars), without sealants or restoration, were examined. The patient sample consisted of 10 women and 11 men with age ranging from 15 to 65 and involved mostly young adult patients in their thirties (Table 3).

### 3.1. ICDAS-II and SOPROLIFE Scores Distribution

The recorded data by each observer are presented in Table 4. Most lesions were noted in the 0 to 2 range of ICDAS-II criteria or in the 0 to 5 range of SOPRO blue fluorescence codes.

### 3.2. Interobserver Reproducibility

The reproducibility of measurements by each observer was first calculated by the means of ICC for each observer and for each diagnostic method (ICDAS-II and SOPROLIFE) as shown in Table 5. The level of interobserver agreement was found to be high both for visual ICDAS-II scored examination (ICC = 0.972; *P* < 0.001) and for SOPROLIFE (ICC = 0.979; *P* < 0.001).

### 3.3. Agreement between ICDAS-II and SOPROLIFE Methods

Since each observer examined and scored the same teeth by both ICDAS-II and SOPROLIFE, the means of the two measurements done by each observer were calculated for each tooth and diagnostic method and used to determine agreement and reliability between the two methods. The reliability between methods was computed using the intracoefficient correlation scale; here we considered the ICDAS-II and SOPROLIFE scales as quantitative variables. Means values for each method (ICDAS II mean = 1.69 ± 1.48 and SOPROLIFE mean = 1.56 ± 1.52) did not differ significantly and a high intraclass correlation coefficient was found (ICC = 0.92; CI = 0.901–0.940; *P* < 0.001). Thus, our results showed a high agreement between the two methods of caries detection.

**Figure 1:**
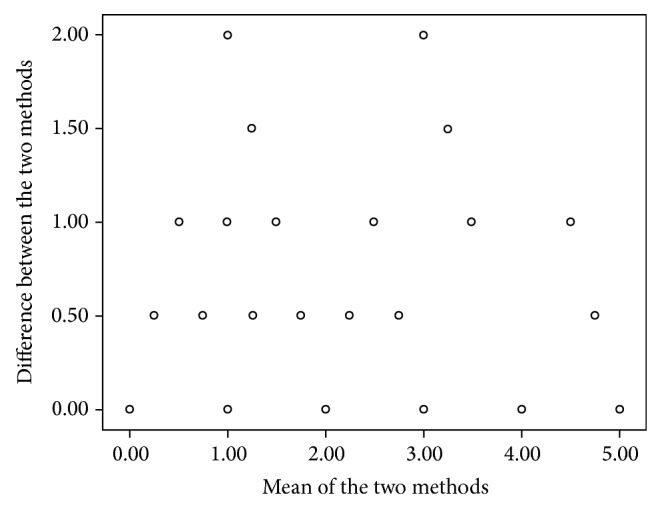
Bland-Altman analysis.

Figure 1 shows the Bland-Altman analysis. The *x*-axis shows the mean of the results of the two methods ([SOPRO + ICDAS-II]/2), whereas the *y*-axis represents the absolute difference between the two methods ([SOPRO − ICDAS-II]). Our results showed an acceptable discrepancy between methods (Figure 1).

### 3.4. Sensitivity and Specificity for the SOPROLIFE

In this study we further attempted to estimate both sensitivity and specificity of the SOPROLIFE blue light irradiation in regard to the visual examination score ICDAS-II used as a reference. Sensitivity was measured as the proportion of actual caries lesions which are correctly diagnosed by SOPROLIFE in regard to ICADS-II, whereas specificity was measured as the proportion of noncarious lesions which were correctly diagnosed by SOPROLIFE in regard to that of ICADS-II. For this purpose, we considered the following two groups: the noncarious (sound tooth surface) lesion group that comprised the 0 scores for each method and the visual change in enamel group that included both score 1 and score 2 groups for each method. These results showed that SOPROLIFE detects noncarious lesions in 88% (specificity measurement) of the cases diagnosed by ICDAS-II. Visual change in enamel was detected by SOPROLIFE in 93% (sensitivity measurement) of the cases detected by ICDAS-II (Table 6).

## 4. Discussion

The present study assessed and compared the newly marketed caries lesion detection tool SOPROLIFE diagnostic mode to the ICDAS-II system.

The results of this study found an almost perfect agreement among the two methods of caries detection with no statistical significant differences between scoring with visual examination and LED fluorescence. This indicates that the diagnosis made with visual examination is roughly the same as the diagnosis made by SOPROLIFE. In addition, according to our results, the number of teeth with 0 score was greater when using fluorescence LED with no statistical difference.

The perfect agreement between the two techniques found in our study has been demonstrated in previous study [13]. The visual examination is routinely used for detecting caries in dental clinics and was also used in recent studies comparing the efficacy of various visual aids. It has the benefit that it is quick and easy to perform, does not need expensive equipment, and can be completed without unnecessary radiation or fluorescence [14]. On the other hand, in the results from* in vitro* study conducted to determinate which nondestructive diagnostic method is clinically applicable and reliable at resolving early enamel changes in occlusal fissure caries created in the laboratory, SOPROLIFE demonstrated only additional light scattering due to the demineralization process [21].* In vitro* and* in vivo* studies showed that different fluorescence signals emitted by SOPROLIFE were a helpful guide for caries detection and excavation [19].

Despite the clinical comparable results between the two diagnostic methods found in our study and in literature, the visual examination presents many limitations in its use. Indeed, one of its limitations is that it requires subjective evaluations to be made by the practitioner; lesions can go undetected because teeth are typically examined by the naked eye. In addition, studies showed that training dental examiners is an essential component of good quality control in dental research [22]. The examiners should be experienced dentists with an interest in cariology and the teeth should be well cleaned for a better visual examination [23]. Stained sites, areas of fluorosis, or developmental defects could be incorrectly scored as caries [9]. By meticulously examining clean dry teeth, sensitivity of a visual examination can be improved after a short training period [9]. Furthermore, meticulous visual inspection with a good operation light, a dry tooth, and a probe can render good sensitivity and specificity values [14]. The readings may also be influenced by several factors such as calculus, plaque and prophylactic pastes, and nonconsistent cleaning procedures [12]. Therefore, caries detection by eyesight is better at an advanced stage than early and presents many limitations related to the experience of the examiner and to the preparation procedure of the teeth examined. Consequently, diagnosis of the caries process by visual inspection is partial and auxiliary methods are needed as adjunct to conventional examination for identifying and quantifying such lesions [12, 24]. In addition, one other disadvantage of ICADS-II is that no images can be taken in order to save the findings for longitudinal monitoring.

In contrast, with SOPROLIFE system, the lesion and its real topography can be seen in a magnified enlarged view [13]. Several studies have shown that the additional observation with the SOPROLIFE camera might also prevent unnecessary operative interventions based on high fluorescence scores due to the better visibility [23, 25]. Due to that “visibility” of the lesion, the interpretation of higher fluorescence answers is easier [26]; the observation capacity of the SOPROLIFE system should guide the clinician toward a more preventive and minimally invasive treatment strategy with monitoring lesion progression or remineralisation over time and not tempt him/her to overtreat a lesion [27].

When comparing the measurements between the two examiners for both methods, our results demonstrated a high reproducibility among the two methods of diagnosis. These results indicated similarity in diagnosis among the 2 observers with both techniques. Despite the different degrees of experience in detecting caries between the two observers, this high interobserver agreement could result from the fact that the observers were from the same department and had a suitable training and calibration session before starting teeth's examination.

In the current study, ICDAS-II was set as “gold standard” [14] due to validated relationship between its codes and the histological depth of a carious lesion as in many other studies [13, 28]. In addition, several studies have shown good reproducibility and accuracy of ICDAS-II for occlusal caries detection in permanent teeth [29] especially caries lesions in the outer half of the enamel [29].

Our results show a high sensitivity and specificity of SOPROLIFE blue fluorescence mode consistently with other studies [13], probably due to its better visibility.

Finally sensitive caries diagnostic tools can serve not only for early detection but also for monitoring of caries lesions to confirm the success of prevention and remineralisation efforts. In order to limit diagnostic errors resulting not only from failure to detect caries, but also from unnecessary preparing of healthy fissures, it is vital to enhance the visual examination (ICDAS-II method) with other sensitive and specific methods as the SOPROLIFE system.

## 5. Conclusion

Compared to the most used visual method in the diagnosis of occlusal caries lesions, the finding from this study suggests that SOPROLIFE can be used as a reproducible and reliable assessment tool. At a cut-off point, categorizing noncarious lesions and visual change in enamel, SOPROLIFE shows a high sensitivity and specificity. We can conclude that financially ICDAS is better than SOPROLIFE. However SOPROLIFE is easier for clinicians since it is a simple evaluation of images. Finally in terms of efficiency SOPROLIFE is not superior to ICDAS but tends to be equivalent with the below advantages.

- High-resolution fluorescence images are likely to provide reliable scores. The better visibility of such images could prevent unnecessary operative intervention.
- We can compare images (before and after).

SOPROLIFE may suffer from interference since it is light based. It might also give false positive results if images are magnified above a certain threshold. Both effects are not elaborated within this study.

## Figures and Tables

**Table 1:** International caries detection and assessment system criteria used in visual examination [11].

Six-point scale categories	Criteria	Clinical lesions
0	Sound tooth surface.	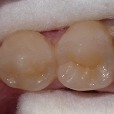

1	First visual change in enamel.	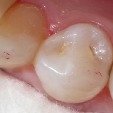

2	Distinct visual change in enamel.	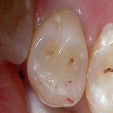

3	Microcavitation in enamel.	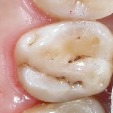

4	Underlying dark shadow from dentine with or without cavitation.	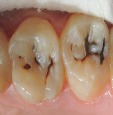

5	Distinct cavity with visible dentine.	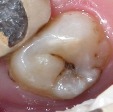

6	Extensive distinct cavity with visible dentine.	

**Table 2:** Scores of SOPROLIFE in blue fluorescence mode [13].

Five-point scale categories	Criteria	Clinical lesions
0	Fissure appears as shiny green; enamel appears sound. A graphite-pencil-colored thin shine/line is rarely observed.	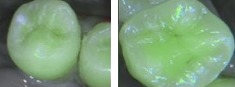

1	Tiny, thin red shimmer in the pit and fissure system is viewed. No red dots appeared.	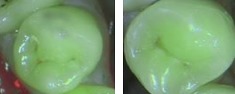

2	In addition to tiny, thin red shimmer in pits and fissures possibly coming up the slopes darker red spots confined to the fissure are visible. There was no surface roughness.	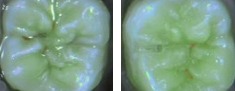

3	Dark red extended areas are confined to the fissures. Slight roughness is possible.	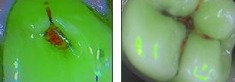

4	Dark red areas are wider than fissures. Surface roughness occurs. Possibly grey or rough grey zone may be visible.	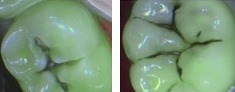

5	Obvious enamel breakdown with visible dentine was observed.	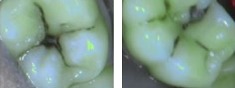

**Table 3:** Characteristics of the study population.

Characteristics	*N* (%) or mean (SD)
Patients age	30.61

Male patients	11 (52.4%)
Female patients	10 (47.6%)

Permanent molars	98 (44.7%)
Permanent premolars	121 (55.3%)

**Table 4:** Distribution of the ICDAS-II and SOPROLIFE blue fluorescence mode scores by both observers.

Method	Observer	0 (*n*)	1 (*n*)	2 (*n*)	3 (*n*)	4 (*n*)	5 (*n*)	6 (*n*)
ICDAS-II score (*n* = 219)	1	56	56	61	16	10	20	—
	2	45	59	60	20	10	21	—

SOPROLIFE blue fluorescence score (*n* = 219)	1	51	57	64	16	10	21	—
	2	68	52	60	11	7	21	—

**Table 5:** Interobserver repeatability among the two observers.

Type of examination	ICC∗ (CI^†^ 95%)
ICDAS-II	0.972^‡^ (0.964–0.979)
SOPROLIFE	0.979^‡^ (0.972–0.984)

^*^ICC = intraclass coefficients.

^†^CI = confidence interval.

^‡^ *P* value < 0.001.

**Table 6:** Validity of the SOPROLIFE regarding ICDAS-II.

Tools	Group 1∗ % (*n*) of teeth scored as noncarious and non cavitated	Group 2^†^ % (*n*) of teeth scored as carious and non cavitated	Sensitivity^‡§b^	Specificity^‡||b^
ICDAS-II	25.5 (56)	53.3 (117)		
SOPROLIFE	29 (64)	52 (114)	93%	88%

^*^Group 1: including score 0.

^†^Group 2: including scores 1 and 2.

^‡^Sensitivity and specificity calculated by taking the ICDAS-II as a gold standard.

^§^Sensitivity measured the proportion of actual caries lesions which are correctly diagnosed by SOPROLIFE regarding ICADS-II.

^||^Specificity measured the proportion of noncarious lesions which are correctly diagnosed by SOPROLIFE regarding ICADS-II.

^b^True negative results = 46; true positive results = 104; false negative results = 8; false positive results = 6.
